# ScRNAbox: empowering single-cell RNA sequencing on high performance computing systems

**DOI:** 10.1186/s12859-024-05935-y

**Published:** 2024-10-01

**Authors:** Rhalena A. Thomas, Michael R. Fiorini, Saeid Amiri, Edward A. Fon, Sali M. K. Farhan

**Affiliations:** 1 Department of Neurology and Neurosurgery, Montreal Neurological Institute-Hospital, McGill University, Montreal, QC H3A 2B4 Canada; 2 The Neuro Early Drug Discovery Unit, Montreal Neurological Institute-Hospital, McGill University, Montreal, QC H3A 2B4 Canada; 3 Department of Human Genetics, Montreal Neurological Institute-Hospital, McGill University, Montreal, QC H3A 2B4 Canada

**Keywords:** Single-cell RNA sequencing, ScRNAseq, High performance computing systems, Pipeline

## Abstract

**Background:**

Single-cell RNA sequencing (scRNAseq) offers powerful insights, but the surge in sample sizes demands more computational power than local workstations can provide. Consequently, high-performance computing (HPC) systems have become imperative. Existing web apps designed to analyze scRNAseq data lack scalability and integration capabilities, while analysis packages demand coding expertise, hindering accessibility.

**Results:**

In response, we introduce scRNAbox, an innovative scRNAseq analysis pipeline meticulously crafted for HPC systems. This end-to-end solution, executed via the SLURM workload manager, efficiently processes raw data from standard and Hashtag samples. It incorporates quality control filtering, sample integration, clustering, cluster annotation tools, and facilitates cell type-specific differential gene expression analysis between two groups. We demonstrate the application of scRNAbox by analyzing two publicly available datasets.

**Conclusion:**

ScRNAbox is a comprehensive end-to-end pipeline designed to streamline the processing and analysis of scRNAseq data. By responding to the pressing demand for a user-friendly, HPC solution, scRNAbox bridges the gap between the growing computational demands of scRNAseq analysis and the coding expertise required to meet them.

**Supplementary Information:**

The online version contains supplementary material available at 10.1186/s12859-024-05935-y.

## Background

In recent years, single-cell RNA sequencing (scRNAseq) technology has led to remarkable breakthroughs in our understanding of biology, enabling us to explore gene expression at the resolution of individual cells. With technological advancements, we have transitioned from analyzing a few cells to thousands and even hundreds of thousands of cells in a single experiment [1]. While the potential of scRNAseq is immense, it has brought about complexities and computational demands that have yet to be comprehensively addressed. Many useful web-based applications and graphical user interfaces (GUI) have been developed to analyze scRNAseq [2–8]. However, these tools fall short of an end-to-end solution for scRNAseq data analysis due to their inability to take raw sequencing data as input, a limited availability of modifiable analytical parameters, and impeded analysis replicability due to undocumented parameter selections. R and python packages with excellent user guides have been developed to process scRNAseq data; however, these require extensive programming knowledge [9–13]. Additionally, given that users must manually adapt and implement the code, repeating the process for each sample, this process can be laborious, error-prone, and time-consuming. The need to execute the code locally further exacerbates these issues, limiting researchers to the capabilities of their own computational resources. The scale of modern scRNAseq datasets necessitates the use of high-performance computing (HPC) clusters. Yet, to our knowledge, a comprehensive scRNAseq workflow tailored to HPC environments hitherto has been unavailable.

In response to these multifaceted challenges, we introduce scRNAbox, a novel and robust scRNAseq analysis pipeline meticulously designed for HPC systems. ScRNAbox not only standardizes and simplifies the scRNAseq analysis workflow for geneticists and biologists with any levels of computational expertise, but also diligently documents execution parameters, ensuring transparency and replicability. It has been assembled to be effortlessly scalable, catering to the evolving needs of researchers faced with large-scale datasets. ScRNAbox provides a unified and accessible resource for the growing community of scRNAseq researchers.

Finally, we recognize the shortage of resources that provide best practices in scRNAseq analysis [14, 15]. In this context, we deploy scRNAbox using publicly available data and outline the decisions bioinformaticians must make during analysis to investigate the biology. To illustrate the utility of scRNAbox, we analyze single-nuclei RNA sequencing (snRNAseq) data published by Smajic and colleagues of midbrain tissue from patients with Parkinson’s Disease (PD) and controls [16]. We outline each step in the scRNAbox pipeline, providing the scientific rationale and the analytical decisions taken in processing the data.

## Implementation

### scRNAbox overview

**Fig. 1:**
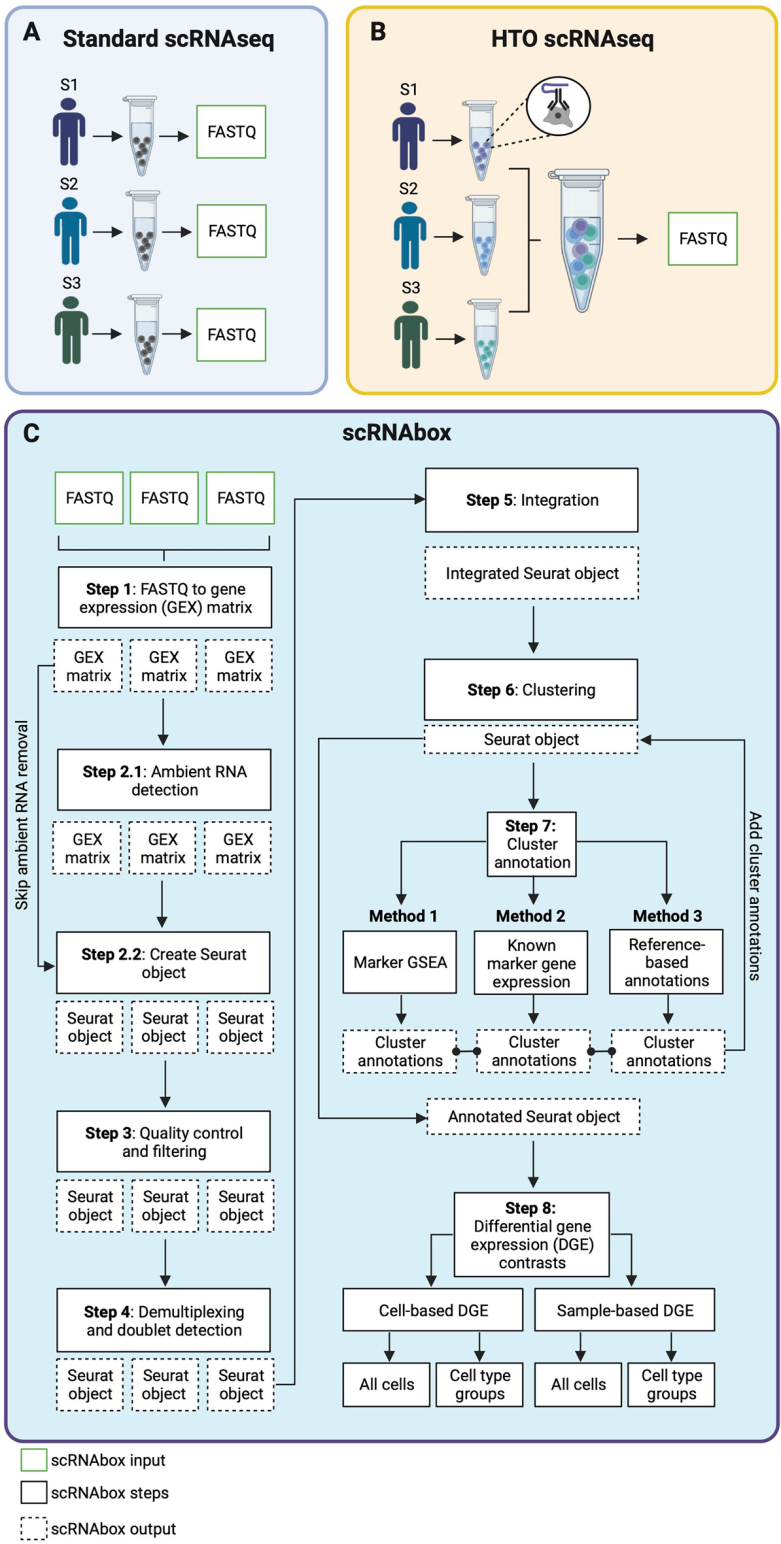
ScRNAbox analysis workflow. The scRNAbox pipeline provides two analysis tracks: 1) standard scRNAseq and 2) HTO scRNAseq. **A** Standard scRNAseq data is prepared by sequencing each sample separately, resulting in distinct FASTQ files for each sample. **B** HTO scRNAseq data is produced by tagging the cells from each sample with unique oligonucleotide “Hashtag” conjugated antibodies (HTO). Tagged cells from each sample are then pooled and sequenced together to produce a single FASTQ file. Sample-specific HTOs are used to computationally demultiplex samples downstream. **C** Steps of the scRNAbox pipeline workflow. Steps are designed to run sequentially and are submitted using the provided bash scripts through the command line. scRNAbox takes FASTQ files as input into Step 1; however, the pipeline can be initiated at any step which takes the users processed data as input

The scRNAbox pipeline consists of R scripts utilizing the Seurat framework, and other R packages including Doublet finder and SoupX for scRNAseq analysis, which are submitted to the SLURM workload manager (job scheduling system for Linux HPC clusters) using bash scripts from the command line [17]. Beginning with 10X Genomics expression data from raw sequencing files, the pipeline facilitates standard steps in scRNAseq processing through to differential gene expression between two different conditions. The scRNAbox framework consists of three main components: (i) R scripts, (ii) job submission scripts, and (iii) parameter and configuration files. The pipeline is separated into Steps, which correspond to analytical tasks in the scRNAseq analysis workflow (Fig. 1). Users can tailor their analysis by manipulating the parameters in the step-specific parameter files. The pipeline can analyze scRNAseq experiments where each sample is captured separately (standard track) or multiplexed experiments where samples are tagged with sample-specific oligonucleotide tagged Hashtag antibodies (HTO), pooled, and sequenced together (HTO track) [18, 19]. The results of each step are reported in intuitive tables, figures, and intermediate Seurat objects [9]. Upon submitting the bash script for a step, “Jobs”, or resource requests are created based on the parameters defined in the configuration file, including CPUs, memory, and time. Jobs are submitted to the HPC system using the SLURM “Scheduler” to execute the R scripts. A complete user guide and the code used in this manuscript can be found at the scRNAbox GitHub site: https://neurobioinfo.github.io/scrnabox/.

### Installation

ScRNAbox can be installed by two different methods on any HPC Linux system. 1) Direct installation via the *scrnabox.slurm* package, which contains the Bash and R scripts, parameter files, and configuration file. The HPC system must have CellRanger (10 × Genomics) and R (v4.2.1 or higher) [20] installed and must use a SLURM scheduler. Users must also run a provided bash script which will automatically install all of the R libraries required for the scRNAbox pipeline. 2) Through a Singularity container, which provides the Bash and R scripts, R library, and Cell Ranger. The container can work with the SLURM scheduler or without job submissions directly on a local computer with sufficient memory. The Singularity container is available from Zenodo: https://zenodo.org/records/12751010.

### Step 0: Initiation and configuration

Following installation, users run Step 0 to initiate the pipeline and specify if they will use the standard or HTO analysis track. Step 0 creates the job submission configuration files and the step-specific parameter files. The configuration file contains the time and memory usage settings for each step and must be edited to match the user’s needs. After Step 0, each subsequent Step can be run individually through separate commands or all together in a single command.

### Step 1: FASTQ to gene expression matrix

#### File structure and inputs

Prior to running the CellRanger *counts* pipeline, a parent directory (“samples_info”) must be created in the working directory. The “samples_info” directory must contain a folder for each sample; the name of the sample-specific folders will eventually be used to name the samples in downstream steps. Each sample-specific folder must contain a *library.csv* file, which defines the information of the FASTQ files for the specific sample. The HTO analysis track also requires a *feature_ref.csv* file, which specifies the oligonucleotide sequences of the Hashtags. Step 1 runs a script to automatically generate these files based on the user input in the parameter file. However, users can manually generate the required files and structure.

#### Running cellranger

ScRNAbox deploys the CellRanger *counts* pipeline to perform alignment, filtering, barcode, and unique molecular identifier counting on the FASTQ files. Each sample is processed by the CellRanger *counts* pipeline in parallel. Although CellRanger is processed with default parameters, all relevant parameters can be adjusted (10X Genomics).

### Step 2: Create Seurat object and remove ambient RNA

#### Ambient RNA detection

The R package SoupX is used to account for ambient RNA, providing users the option to correct the gene expression matrices for RNA contamination [21]. SoupX quantifies the contamination fraction according to the expression profiles of empty droplets and cell clusters identified by the CellRanger *counts* pipeline. Marker genes used to estimate the contamination rate are automatically identified using the *AutoEstCont* function and the expression matrix is corrected per the estimated contamination rate using the *adjustCounts* function.

#### Generation of the seurat object and quality control metrics

The Seurat function *CreateSeuratObject* is used to take in the CellRanger (if not removing ambient RNA) or SoupX (if removing ambient RNA) generated feature-barcode expression matrices, and create the list-type Seurat object [9]. The number of genes expressed per cell (number of unique RNA transcripts) and the total number of RNA transcripts are automatically computed. The proportion of RNA transcripts from mitochondrial DNA (gene symbols beginning with “MT”) and the proportion of ribosomal protein-related transcripts (gene symbols beginning with “RP”) are both calculated using the Seurat *PercentageFeatureSet* function. Following the Seurat workflow, the *CellCycleScoring* function with the Seurat S and G2/M cell cycle phase reference genes are used to calculate the cell cycle phase scores and generate a principal component analysis (PCA) plot [22].

### Step 3: Quality control and generation of filtered data objects

ScRNAbox allows users to filter low quality cells by defining upper- and lower-bound thresholds in the parameter files based on unique transcripts, total transcripts, percentage of mitochondrial-encoded transcripts, and percentage of ribosome gene transcripts. Users can also remove or regress a custom gene list from the dataset. The filtered counts matrix is then normalized, the top variably expressed genes are identified, and the data are scaled using Seurat functions. Linear dimensional reduction is performed via PCA and an elbow plot is generated to visualize the dimensionality of the dataset and inform the number of principal components (PC) to be used for doublet detection in Step 4.

### Step 4: Demultiplexing and doublet removal

#### Doublet detection and removal (Standard track)

Barcodes that are composed of two or more cells are identified as doublets using DoubletFinder [23]. Doublets are predicted based on the proximity of each cell’s gene expression profile to that of artificial doublets created by averaging the transcriptional profiles of randomly chosen cell pairs. The default value of 0.25 for the number of artificial doublets is used. The neighbourhood size corresponding to the maximum bimodality coefficient is selected and the proportion of homotypic doublets is computed using the *modelHomotypic* function. Users can define the number of PCs to use for doublet detection and the expected doublet rate for each sample. Users have the option to remove doublets from downstream analyses or just calculate the doublet rate.

#### Demultiplexing followed by doublet removal (HTO track)

Pooled samples are demultiplexed based on their sample-specific HTO labels using Multi-seq [19]. The automatically detected inter-maxima quantile thresholds of the probability density functions for each barcode are used to classify cells. Cells surpassing one HTO threshold are classified as singlets; cells surpassing > 1 thresholds are classified as doublets; the remaining cells are assigned as “negative”. The counts observed for each barcode are reported in a summary file and plots are generated to visualize the enrichment of barcode labels across sample assignments. Users have the option to remove doublets and negatives from downstream analyses.

### Step 5: Creation of a single Seurat object from all samples

#### Integration or merging samples

The individual Seurat objects are integrated to enable the joint analysis across sequencing runs or samples by deploying Seurat’s integration algorithm [24]. The genes that are variable across all samples are detected by the *SelectIntegrationFeatures* function. Integration anchors (pairs of cells in a matched biological state across datasets) are selected by the *FindIntegrationanchors* function, and the *IntegrateData* function is used to integrate the datasets by taking the integration anchors as input. Alternatively, users may simply merge the normalized counts matrices using Seurat’s *merge* function without performing integration.

#### Linear dimensional reduction

Seurat functions are used to normalize the count matrix, find the most variably expressed genes, and scale the data. Linear dimensional reduction is then performed via PCA using the top variably expressed genes as input. An elbow plot to visualize the variance contained within each PC and jackstraw plot to visualize “significant” PCs are produced. These plots inform the number of PCs that should be retained for clustering in Step 6.

### Step 6: Clustering

Clustering is performed to define groups of cells with similar expression profiles using the Seurat implementation of the Louvain network detection with PCA dimensionality reduction as input [9]. K-nearest neighbours are calculated and used to construct the shared nearest neighbour graph. The Jaccard similarity metric is used to adjust edge weights between pairs of cells, and the Louvain algorithm is used to iteratively group cells together based on the modularity optimization. To assist users in selecting the optimal clustering conditions, we include an option to compute the Louvain clustering *N* times at each clustering resolution, while shuffling the order of the nodes in the graph for each iteration. The average and standard deviation of the Adjusted Rand Index (ARI) between clustering pairs at each clustering resolution is then calculated [25]. A *ClustTree* plot [26] and uniform manifold approximation and projection (UMAP) plots are generated to visualize the effect of clustering parameters.

### Step 7: Cluster annotation

Cluster annotation is performed to define the cell types comprising the clusters identified in Step 6. ScRNAbox provides three tools to identify cell types comprising the clusters.

#### Tool 1: Cluster marker gene identification and gene set enrichment analysis

ScRNAbox identifies genes that are significantly up regulated within each cluster by using the Seurat *FindAllMarkers* function, implementing the Wilcoxon rank-sum test [9] with a log2 fold-change (L2FC) threshold of 0.25. Differentially expressed genes (DEGs) are calculated by comparing each cluster against all other clusters. Only upregulated genes are considered for cluster marker genes. A heatmap is generated to visualize the expression of the top marker genes for each cluster at the cell level. All significant, upregulated DEGs are used as the input for gene set enrichment analysis (GSEA) across user-defined libraries that define cell types using the EnrichR tool [27]. Cluster-specific tables are generated to report all enriched cell types and bar plots visualize the most enriched terms.

#### Tool 2: Expression profiling of cell type markers and module scores

ScRNAseq allows users to visualize the expression of individual genes and the aggregated expression of multiple genes from user-defined cell type marker gene lists. For each gene in a user-defined list, a UMAP plot visualizes its expression at the cell level, while violin and dot plots visualize its expression at the cluster level. Aggregated expression of user-defined cell type marker gene lists is calculated using the Seurat *AddModuleScore* function [22]. The average expression of each cell for the gene set is subtracted from randomly selected control genes, resulting in cell-specific expression scores, with larger values indicating higher expression across the gene set.

#### Tool 3: Cell type predictions based on reference data

ScRNAbox utilizes the Seurat label transfer method: *FindTransferAnchors* and *TransferData* functions, to predict cell-type annotations from a reference Seurat object [24]. Predicted annotations are directly integrated into the query object’s metadata and a UMAP plot is generated to visualize the query dataset, annotated according to the predictions obtained from the reference.

#### Adding annotations

ScRNAbox uses the Seurat *AddMetaData* function and a user-defined list of cell types in the parameter file to add cluster annotations. The cluster annotations from each iteration of the step will be retained, allowing users to define broad cell types and subtypes. UMAP plots with the annotation labels are generated to visualize the clustering annotations at the cell level, allowing users to check the accuracy of their annotations.

### Step 8: Differential gene expression analysis (DGE)

Metadata defining the groups to be compared are added to the Seurat object by submitting a.csv file containing sample information with phenotypic or experimental data. The additional metadata is used to define the variables to compare for the DGE. ScRNAbox allows DGE to be calculated between conditions using all cells or cell type groups using two different data preparations: cell-based or sample-based DGE.

#### Cell-based DGE

Cells are used as replicates and DGE is computed using the Seurat *FindMarkers* function to compare user-defined contrasts for a given variable [9]. While *FindMarkers* supports several statistical frameworks to compute DGE, we set the default method in our implementation to MAST, which is tailored for scRNAseq data [28]. MAST models both the discrete expression rate of all genes across cells and the conditional continuous expression level, which is dependent on the gene being expressed in the cell, by a two-part generalized linear model [28]. Regardless of the method used, P values are corrected for multiple hypothesis testing using the Bonferroni method. Users can perform their own p-value adjustments using the DEG files output from the pipeline.

#### Sample-based DGE

To calculate DGE using samples or subjects as replicates, scRNAbox applies an aggregate pseudo-bulk analysis [29]. First, the Seurat *AggregateExpression* function is used to compute the sum of RNA counts for each gene across all cells from a sample [30]. These values are then input into the DESeq2 framework, which uses gene dispersal to calculate DGE [31]. P-values are corrected for multiple hypothesis testing using the Bonferroni method, which can be recalculated from the pipeline output.

### Analysis of differentially expressed genes

Step 8 produces data tables of the DEGs for each of the defined contrasts. These outputs can be used for gene enrichment pathway analysis using web-apps or though application program interfaces with reference libraries using a programming language, in our case, R. Further analysis of the results is experiment-dependent and must be completely tailored to the research questions. We used the ClusterProfiler R package to identify significantly enriched Gene Ontology (GO) terms with the *gseGO* function [32]. We utilized the 'org.Hs.eg.db' Bioconductor annotation package to access human (*Homo sapiens*) gene annotations for our analysis. The ggplot2 R package was used for data visualization [33].

## Results

To demonstrate the functionality of the scRNAbox pipeline we analyzed a publicly available snRNAseq dataset from the post-mortem midbrains of five patients with PD and six controls prepared by Smajic et al. [16]. To demonstrate scRNAbox’s ability to process multiplexed scRNAseq data, we analyzed a scRNAseq dataset of peripheral blood mononuclear cells (PBMCs) from eight human donors prepared by Stoeckius et al. [18].

### ScRNAbox efficiently processes raw sequencing data and provides quality control measures

We initiated our scRNAbox analysis of the midbrain dataset by running Step 0 and selecting the standard track. This created the job configuration file and step-specific parameter files. In Step 1, we used the automatic library preparation function to generate the sample-specific *library.csv* files and ran CellRanger (v5.0.1) *counts* on all 11 subjects.

**Fig. 2:**
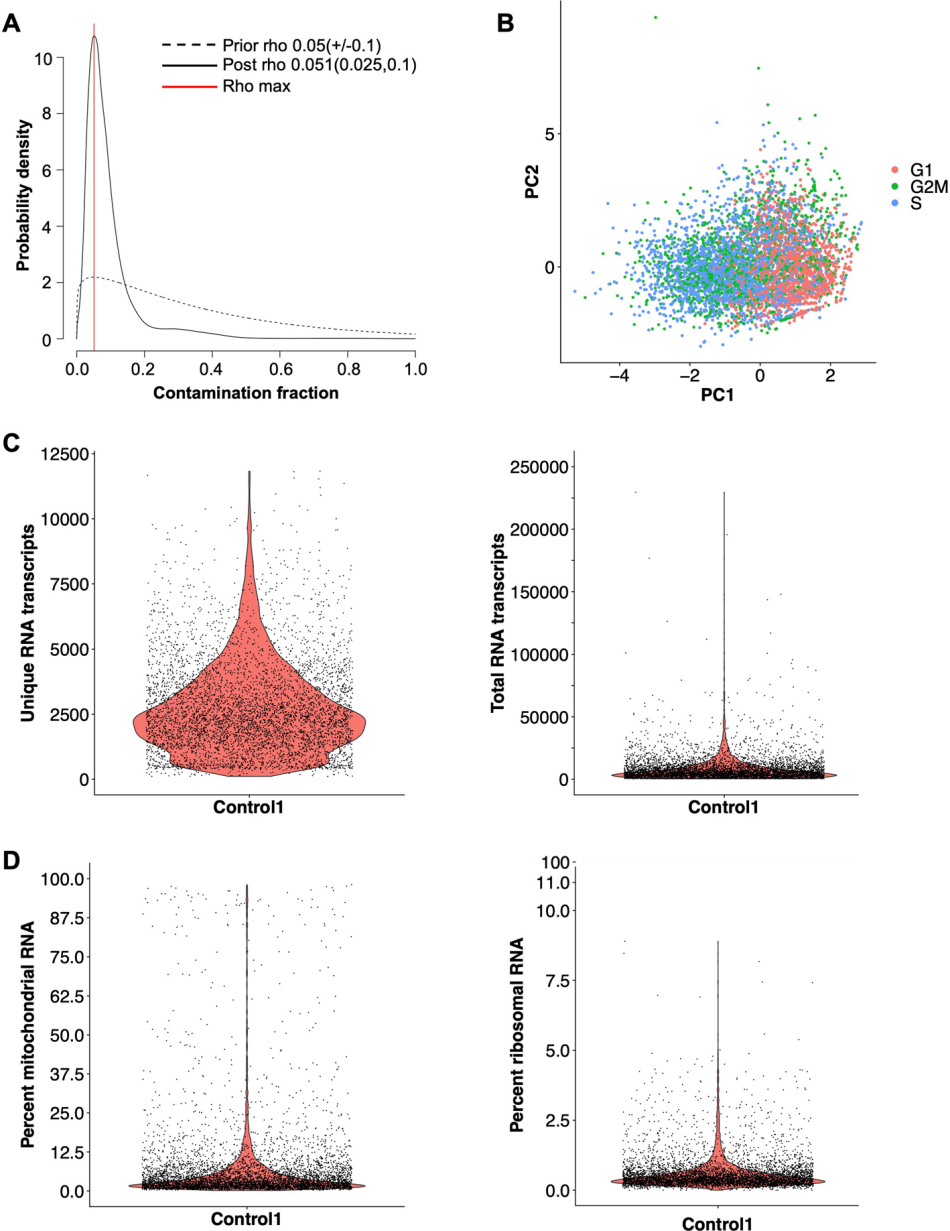
scRNAbox calculates and visualizes quality control metrics.** A** Line plot of the ambient RNA contamination rate (rho) estimated by SoupX [21]. Estimates of the RNA contamination rate using various estimators are visualized via a frequency distribution; the true contamination rate is assigned as the most frequent estimate. The ambient RNA rate for snRNAseq midbrain sample *Control 1* is indicated by the red line (5.1%). **B** Principal component analysis (PCA) of *Control 1* coloured by cell-cycle scores calculated using the Seurat S and G2/M reference genes [22]. **C** Violin plots showing the distribution of RNA transcripts, including unique RNA transcripts per cell (left) and total RNA transcripts per cell (right) in *Control 1*. Individual cells are shown. **D** Violin plots showing the proportion of mitochondrial-encoded RNA (left) and ribosomal RNA (right) in Control 1. Individual cells are shown

In Step 2, the pipeline generates a Seurat object for each sample and computes multiple quality control metrics that inform decisions for filtering in Step 3. At this stage, we had the option to remove ambient RNA, transcripts from an external source captured with a true cell. These aberrant transcripts originate from many possible sources including cells that ruptured or died during dissociation and released their RNA, mRNA-containing exosomes, or mRNA that leaked out when cell processes were cleaved during dissociation. Large amounts of ambient RNA confound the data, making cells appear to have similar transcriptional profiles when they are truly distinct. Leveraging SoupX to detect ambient RNA revealed low contamination rates across all samples (mean = 2.46%) (Fig. 2A**; **Table 1**)**. Cell cycle stage is another quality control metric to consider during scRNAseq data processing as it can affect cell type annotations in downstream analyses. ScRNAbox computes the cell cycle stage for each cell and generates a PCA plot to visualize the effect of cell cycle stage in the data. The cell cycle stage showed little effect on cell distributions in PCA space (Fig. 2B; Supplementary Figure S1).

**Table 1:** Selected quality control measurements across all samples in the midbrain dataset

	Control						Parkinson’s Disease				
Sample	1	2	3	4	5	6	1	2	3	4	5
Ambient RNA rate (%)	5.1	1.9	2.0	2.2	4.3	1.6	2.2	2.3	2.5	1.0	2.0
Minimum total RNA transcripts	501	500	506	500	501	501	500	504	503	501	501
1st quartile total RNA transcripts	2781	2913	2278	3375	2572	3351	2582	3092	2025	2668	3654
Minimum unique RNA transcripts	112	68	199	132	50	154	184	66	261	32	196
1st Quartile unique RNA transcripts	1478	1587	1346	1766	1490	1725	1448	1711	1276	2520	1896
Median % mitochondrial RNA	3.04	1.30	0.62	0.44	1.20	1.02	1.12	1.80	0.55	2.14	0.92
3rd Quartile % mitochondrial RNA	6.19	3.22	1.24	0.89	2.34	2.30	2.31	3.42	1.14	4.49	1.69
Median % ribosomal RNA	0.42	0.72	0.64	0.67	0.61	0.76	0.45	0.66	0.79	0.85	0.63
3rd Quartile % ribosomal RNA	0.65	1.16	0.88	1.00	0.87	1.22	0.73	1.14	1.25	1.31	1.13

To further visualize the data and determine thresholds for filtering, scRNAbox computes the unique RNA transcripts and total counts of RNA for each cell (Fig. 2C). Cells with too few unique RNA transcripts are only ambient RNA, membrane fragments, or damaged/dying cells, and these barcodes should be removed. The range of unique transcripts varies across species, tissue types, and sample preparations. The distribution of unique RNA transcripts and total RNA varied across the 11 samples; however, the lowest quartile (1st quartile) value was above 1000 in both measures for all samples, indicating that a stringent threshold for good quality cells will retain a large sample size (Table 1). Finally, the percentage of mitochondrial and ribosomal RNA transcripts are calculated (Fig. 2D). A high proportion of mitochondrial-encoded RNA indicates that the mitochondria are damaged within that cell, indicating that the cell is likely dying. In most cases, researchers will remove these cells. Ribosomal RNA genes encode proteins for ribosomal machinery and indicates a high level of translational activity in the cell. Like cell cycle state, elevated levels of ribosomal proteins could later impact clustering results; however, both may also represent biologically relevant signals that researchers may wish to retain and further explore. As expected from nuclear sequencing, the percentage of mitochondrial-encoded genes was low across all samples (Table 1).

### ScRNAbox applies quality control filters and integrates samples

**Fig. 3:**
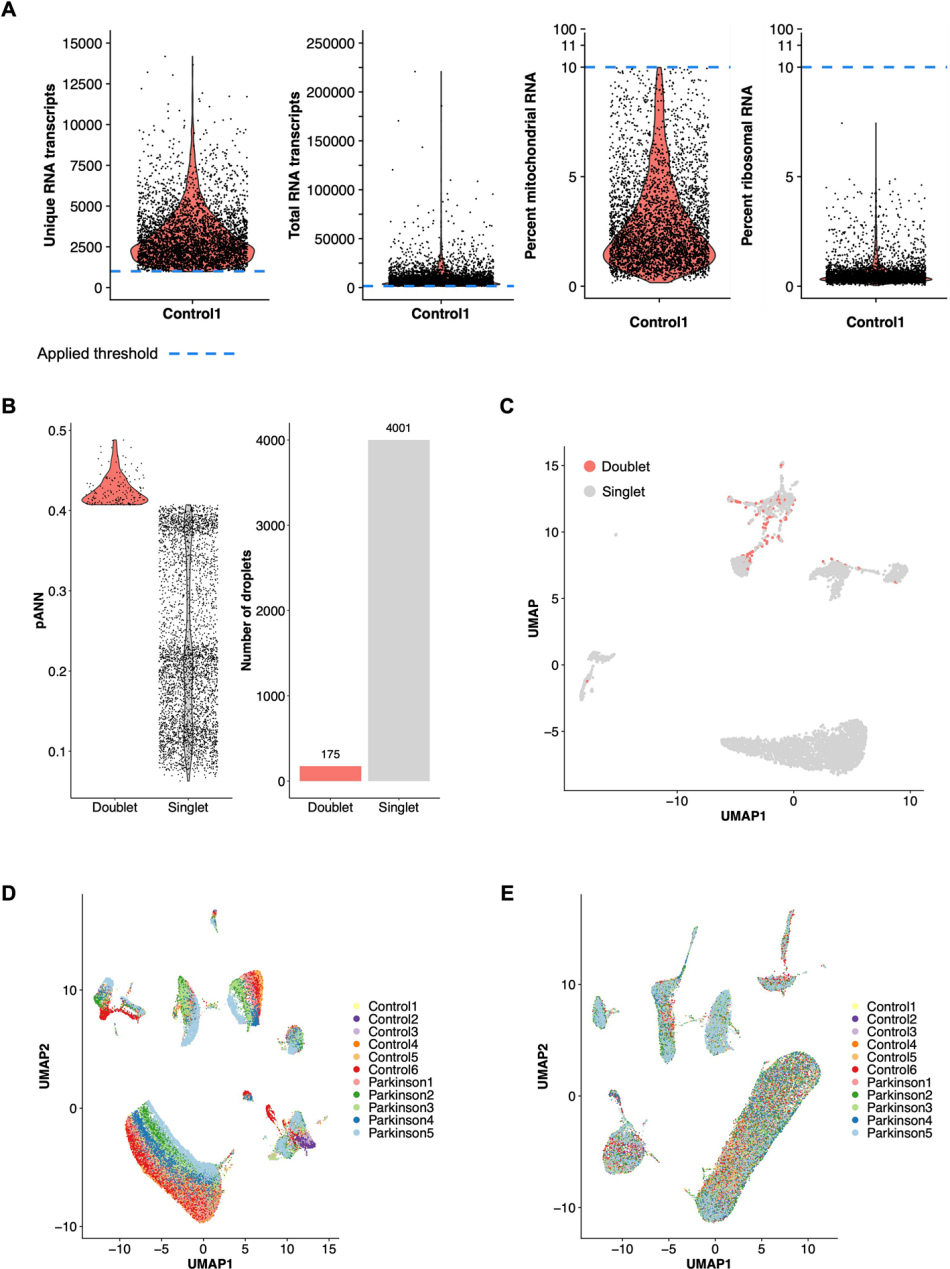
scRNAbox produces visualizations of filter applications, doublet detection, and data integration. **A** Violin plots visualizing the distribution of quality control metrics after filtering according to user-defined thresholds, for snRNAseq midbrain sample *Control 1*. **B** Results of doublet detection with DoubletFinder [23]. Left: violin plot displaying the distribution of the proportion of artificial nearest neighbours (pANN) across singlets and doublets for *Control 1*. Right: a bar plot of the number of predicted singlets and doublets for *Control 1*. **C** Uniform Manifold Approximation Projection (UMAP) plots coloured by droplet assignments (singlet or doublet) for *Control 1*. **D** UMAP of merged snRNAseq midbrain samples (six Control and five PD) coloured by sample identity. **E** UMAP of the same data after integration, coloured by sample identity

In step 3, we applied the filtering criteria used by Samjic et al. [16]; we did not adjust for ambient RNA contamination or regress cell cycle genes. We removed unwanted barcodes as described above, applying filters for minimum unique RNA transcripts (> 1000), minimum total RNA transcripts (> 1500), and maximum percent mitochondria and ribosomal RNA (< 10) (Fig. 3A). Additionally, we removed mitochondrial-encoded and ribosomal genes. After applying these filters, we retained between 2,442 and 6,153 cells per sample (Table 2). In Step 4, we leveraged DoubletFinder to predict doublets using default parameters and 25 PCs, and defined the expected doublet rate for each sample based on the number of recovered cells from the CellRanger pipeline (Figs. 3B and 3C**; **Table 2). The DoubletFinder algorithm requires that UMAP dimensional reduction is performed prior to analysis. We performed dimensional reduction using 25 PCs and 65 nearest neighbours. After removing predicted doublets, 44,538 cells remained across all samples. In total, 9,460 cells (17.52%) were filtered from the dataset (Table 2).

**Table 2:** Unique barcode counts at different stages of data processing

Sample	Number of Unique Barcodes	Cell count after filtering	Expected doublet rate (%)	Number of doublets detected	Cell count after doublet removal
Control 1	5513	4176	4.2	175	4001
Control 2	5508	4553	4.2	191	4362
Control 3	2856	2490	2.3	57	2433
Control 4	5347	4952	4.0	198	4754
Control 5	3564	3196	2.7	86	3110
Control 6	7174	6153	5.3	326	5827
PD 1	2899	2442	2.3	56	2386
PD 2	7169	6388	5.3	339	6049
PD 3	4560	3784	3.4	129	3655
PD 4	2820	2307	2.3	53	2254
PD 5	6588	6007	5.0	300	5707
Total	53,998	46,448	NA	1910	44,538

*PD*, Parkinson’s disease

Finally, after processing each individual sample, we combined all samples into one data object to facilitate integrated analysis. In Step 5, users have the option to either merge (Fig. 3D) or integrate (Fig. 3E) the data. We proceeded with downstream analyses of the midbrain dataset using the integrated data object, which facilitates the identification of cell types that are consistent across samples [24].

### ScRNAbox provides tools to optimize clustering and facilitate annotation

**Fig. 4:**
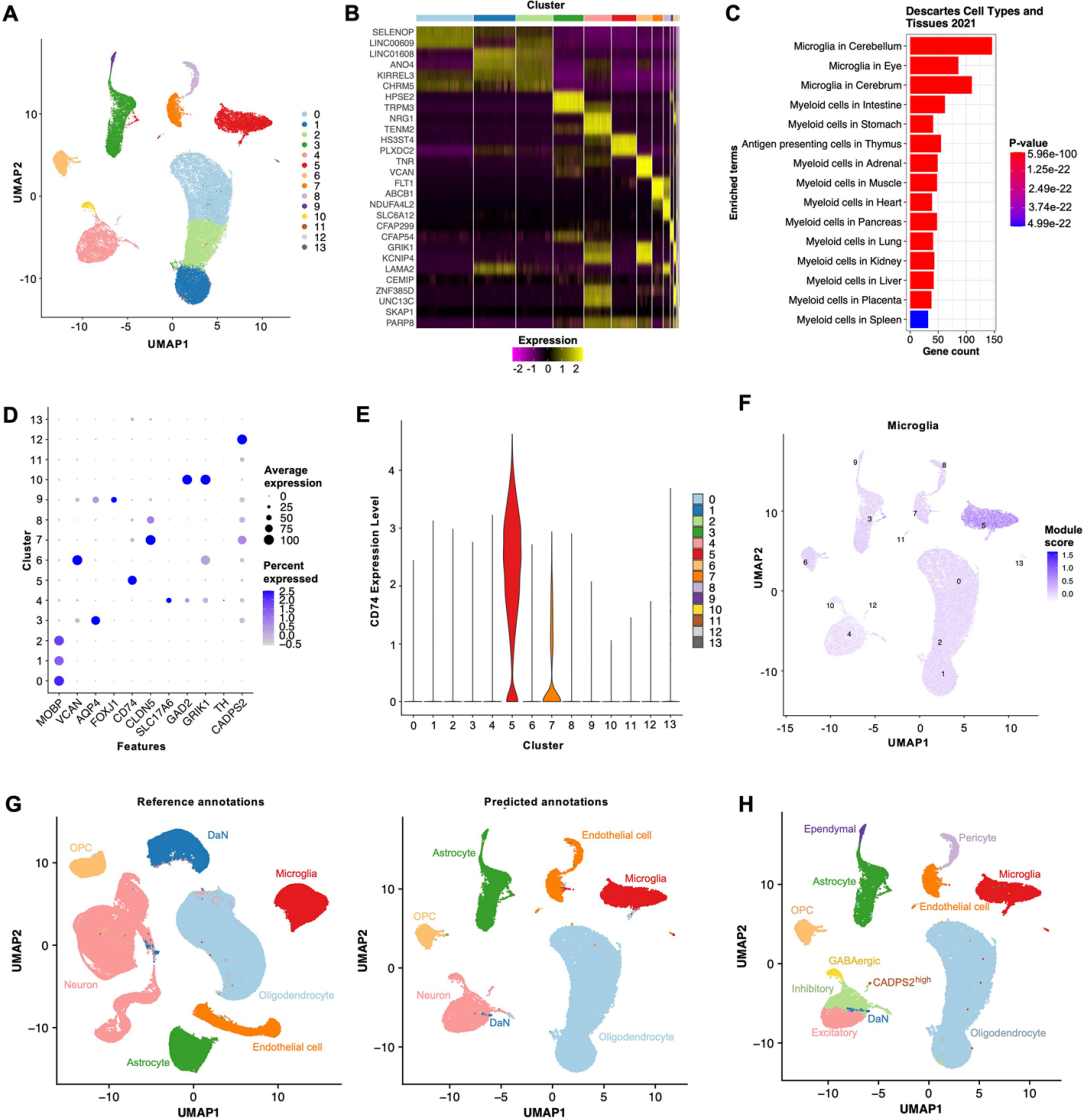
scRNAbox performs clustering to identify cells type groups and provides tools for cluster annotation. **A** Uniform Manifold Approximation Projection (UMAP) plots showing clusters identified by Louvain network detection with a resolution of 0.2, coloured by cluster index. The UMAP was generated from the 11 integrated snRNAseq midbrain samples. **B** Heatmap of the top 3 upregulated marker genes for each cluster in A. **C)** Bar chart showing the top 15 cell types in the *Descartes Cell Types and Tissue* library identified by GSEA of the marker genes for cluster 5. **D** Dot plot showing expression of cell type markers defined by Smajic et al. for each cluster at a clustering resolution of 0.2. The cell type markers are as follows: oligodendrocytes: *MOBP*; oligodendrocyte precursor cells (OPC): *VCAN*; astrocytes: *AQP4*; ependymal cells: *FOXJ1*; microglia: *CD74*; endothelial cells: *CLDN5*; pericytes: *GFRB*; excitatory neurons: *SLC1746*; inhibitory neurons: *GAD2*; GABAergic neurons: *GAD2* and *GRIK1*; dopaminergic neurons (DaN): *TH*. PD specific DaN subgroup; *CADPS2*. **E** Violin plot showing expression levels in each cluster across individual cells for the microglia marker *CD74*. **F** UMAP showing the module score for the microglia gene marker list. The module score is an aggregated expression of known marker genes [22]. **G** Left: UMAP of clustered and annotated reference Seurat object: snRNAseq of midbrain tissue produced by Kamath et al. [34], coloured by cell type. Using the Seurat label transfer approach, the reference data was used to predict cell types in the query data:11 snRNAseq midbrain samples from Smajic et al. [16]. Right: UMAP of the label transfer predictions for each cell, coloured by predicted cell type. **H** UMAP of the 11 integrated samples with the applied final cell type annotation, coloured by cell type

In Step 6, we performed clustering on the integrated dataset to eventually identify distinct cell types. We clustered the cells using the 4000 most variably expressed features and 25 PCs, maintaining the parameters used by Smajic et al. [16]. We used 30 neighbours to construct the shared nearest neighbour graph input into the Louvain network detection algorithm and performed clustering on a range of clustering resolutions (Supplementary Figure S2A). To evaluate the reproducibility of clusters identified at each resolution, we calculated the ARI between clustering pairs at each resolution across 25 replications [25]. The ARI at a clustering resolution of 0.05 and 0.2 were both 1.00 and the ClusTree plot suggested high stability (Supplementary Figures S2B and S2C). Thus, we used a clustering resolution of 0.2, which identified 14 clusters, to annotate the major cell types (Fig. 4A).

In Step 7, we applied the three cluster annotation tools within the scRNAbox pipeline to identify the cell types. Using Tool 1, we identified the top markers for each cluster (Fig. 4B) and subjected these genes to GSEA using the EnrichR R package. As an example, the *Descartes Cell Types and Tissues 2021* library GSEA suggested that cluster 5 are microglia (Fig. 4C). For Tool 2, we profiled the expression of known marker genes, using the marker genes identified by Samjic et al. to annotate their clusters: oligodendrocytes: *MOBP*; oligodendrocyte precursor cells (OPC): *VCAN*; astrocytes: *AQP4*; ependymal cells: *FOXJ1*; microglia: *CD74*; endothelial cells: *CLDN5*; pericytes: *GFRB*; excitatory neurons: *SLC1746*; inhibitory neurons: *GAD2*; GABAergic neurons: *GAD2* and *GRIK1*; dopaminergic neurons (DaN): *TH*. Except for clusters 11 and 13, we found that each cluster showed elevated expression for at least one marker gene (Fig. 4D). ScRNAbox also allows expression profiling of known marker genes through a violin plot. For instance, we explored the expression of *CD74* across clusters and found that cluster 5 showed elevated expression of this gene, further suggesting that this cluster consists of microglia (Fig. 4E). Next, we computed the module scores for custom gene marker lists (Supplementary Table S1). The module score for the microglia gene set was highest in cluster 5 **(**Fig. 4F). Using Tool 3, we predicted cell types using a labelled Seurat object generated from snRNAseq midbrain data published by Kamath et al. [34] (Fig. 4G).

Performing cluster annotations at a clustering resolution of 0.2 allowed us to identify the major cell types expected in the human midbrain. However, to further classify the neurons into subtypes, we repeated Step 7 at a clustering resolution of 1.5, as used by Smajic and colleagues [16]. We subjected the 33 clusters identified to marker GSEA and profiled the expression of known marker genes and cell type marker gene lists (Supplementary Figures S3-6). In doing so, we identified each of the expected neuronal subtypes, including a cluster of rare CADPS2^high^ DaNs identified by Smajic et al., resulting in 12 cell types for our final annotation (Fig. 4H; Supplementary Figure S6D).

### ScRNAseq efficiently calculates differential gene expression and facilitates pathway analysis

**Fig. 5:**
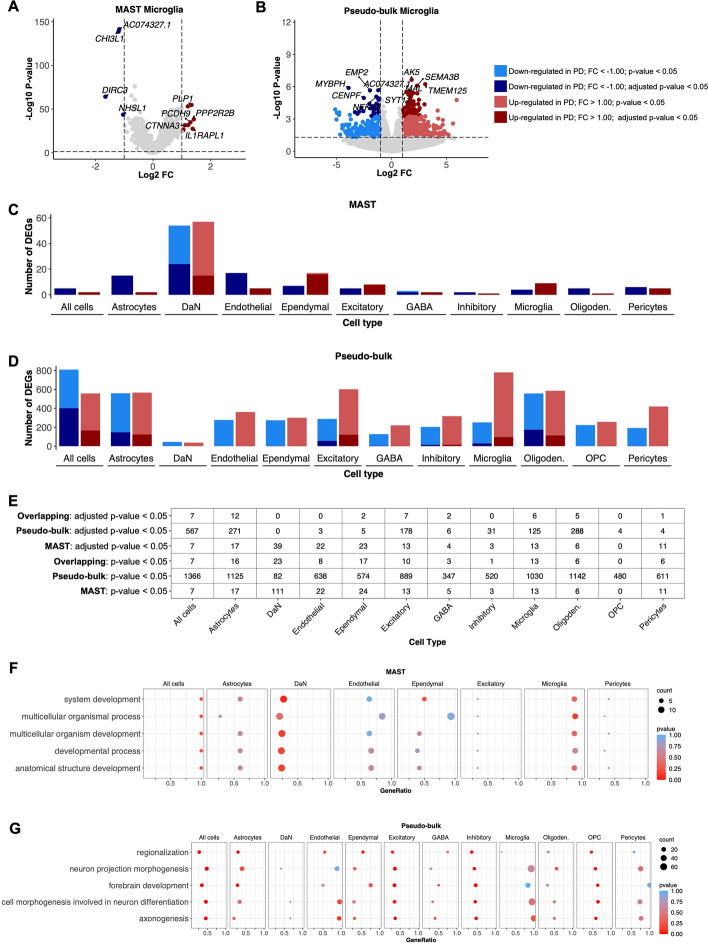
scRNA calculates differential gene expression (DGE) using multiple statistical frameworks. ScRNAbox computes DGE using two distinct data preparations: 1) using cells as replicates and the MAST statistical framework [28] and 2) using samples as replicates (pseudo-bulk) and the DESeq2 statistical framework [31]. **A** Volcano plot showing cell-based DGE results identified by MAST, between Parkinson’s disease (PD) and control subjects for microglia. **B** Volcano plot showing sample-based DGE identified by DESeq2 between PD and control subjects for microglia. **C, D** Bar chart showing the number of differentially expressed genes (DEGs) identified with a an absolute value log2 fold-change (L2FC) > 1 and p-value < 0.05. Bonferroni adjusted p-values < 0.05 are indicated by the darker shades. **C)** Cell-based DGE using MAST. **D** Sample-based DGE using DESeq2 **E** Number of DEGs identified by cell-based DGE with MAST, sample-based DGE with DESeq2, or both frameworks across all cell types. Only DEGs with an absolute value L2FC > 1 are included. **F, G** Bar chart showing the top 5 most enriched GO t-Biological Processes calculated for all cell types together. DEGs with p-values < 0.05 and L2FC > 1 were used as the input for gene set enrichment analysis (GSEA). The gene ratio, gene count, and p-value of the 5 terms in each cell type are shown. **F** GO analysis of DEGs identified by cell-based DGE across all cell types. The missing cell types did not have enough DEGs for GSEA analysis to return results and were not plotted. **G** GO analysis of DEGs identified by sample-based DGE across all cell types

In Step 8, scRNAbox calculates DGE by two different methods, cell-based using MAST [28] — whereby individual cells are used as replicates — and sample-based using DESeq2 [30] [31] — whereby the aggregated counts of individual subjects are used as replicates. Importantly, the choice between statistical frameworks for DGE analysis must be carefully considered by the user. While multiple benchmarking studies have assessed the performance of different methods for DGE analysis, an agreement on the best approach has yet to be obtained as different frameworks perform variably across statistical performance metrics [29, 35]. To perform DGE analysis, we first added metadata to the Seurat object and classified each sample as either “Control” or “PD”, allowing us to define our desired contrasts. Next, we computed DGE between PD and controls for all cells together and for each cell type individually. Cell-based DGE resulted in fewer DEGs with L2FC greater than 1.0 but more genes significant after p-value adjustment for multiple comparisons (Fig. 5A and 5B; Supplementary Figure S8 and S9; Supplementary Table S2 and S3). For example, cell-based-DGE identified 13 DEGs (p-value < 0.05; absolute value L2FC > 1) between PD and controls for microglia, while pseudo-bulk with DESeq2 identified 1,030 DEGs at the same significance threshold and for the same cell type (Fig. 5A and 5B). Indeed, the sample-based-DGE identified a higher number of DEGs across all cell types compared to MAST, except for DaNs (sample-based = 82 DEGs; cell-based = 111 DEGs) (Fig. 5C and 5D). Another benefit of using multiple statistical frameworks for computing DGE is the ability to identify consensus signals. Particularly, the DEGs that maintain significance after correction for multiple hypothesis testing by multiple statistical frameworks may be of highest interest to investigators (Fig. 5E). Finally, the DGE data tables produced by the scRNAbox pipeline can be used to perform gene enrichment pathway analysis and explore the contribution of different cell types to perturbed pathways. As an example, we performed a "GO Biological Processes" analysis using significant DEGs (p-value < 0.05 and L2FC > 1) identified by sample-based DGE [1366 genes] and cell-based DGE [7 genes] upon comparing all cells between PD and control subjects [32]. We then selected the top 5 most significantly enriched pathways from each DGE  method and looked at the pathway significance of each GO term across cell types **(**Fig. 5F and 5G) Interestingly, both DGE methods suggested perturbed pathways related to developmental and neuro-anatomical changes in the PD midbrain.

### ScRNAbox effectively demultiplexes cells with Hashtag feature labels

**Fig. 6:**
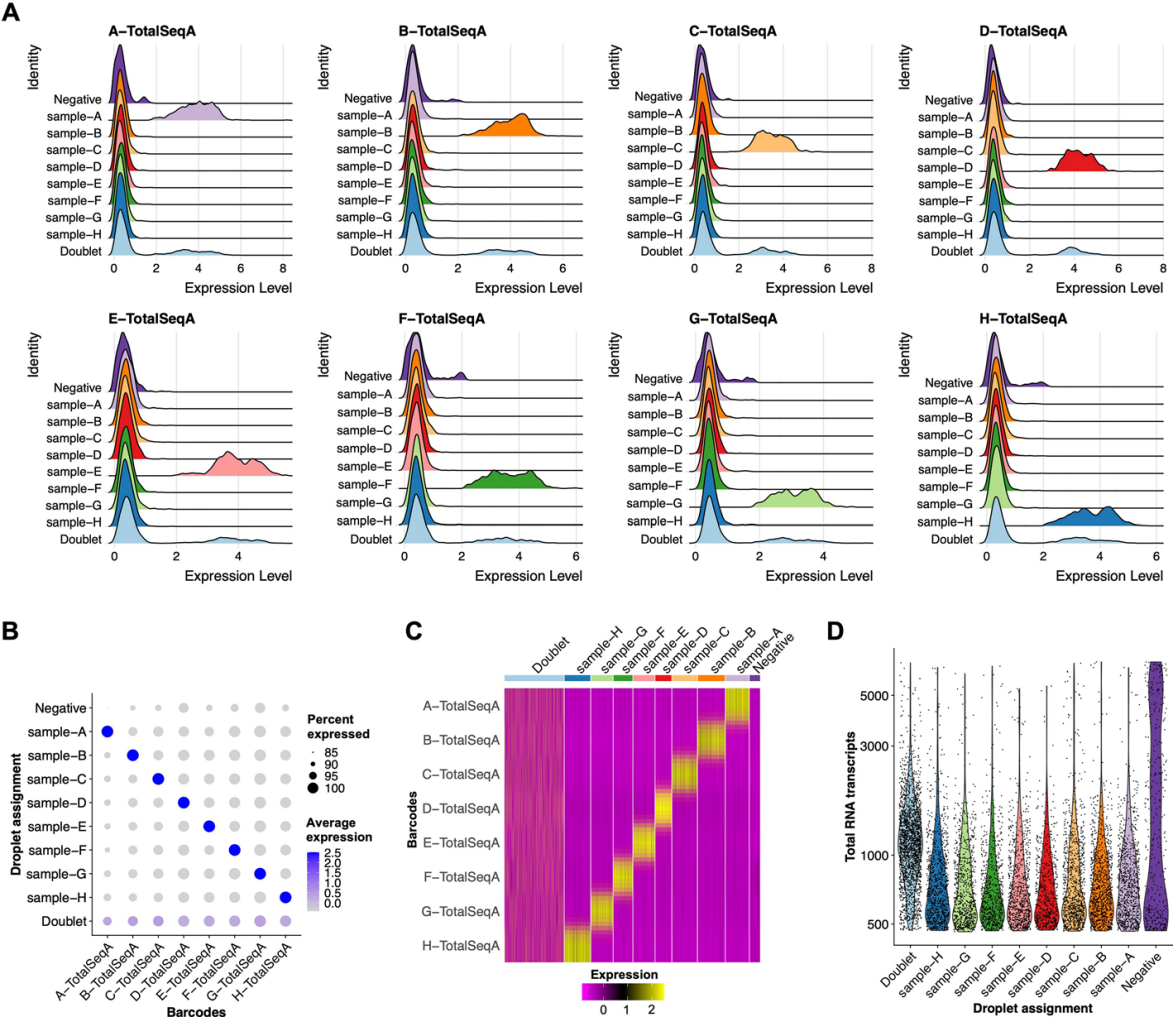
scRNAseq effectively demultiplexes HTO samples and detects doublets. The expression matrices of sample-specific oligonucleotide conjugated antibodies (HTO) are used to demultiplex samples and identify doublets [19]. The enrichment of barcode labels across sample assignments are visualized at the cellular and sample level. **A** Ridge plots (stacked density plots) showing the expression of each HTO tag expression in each assigned sample. **B** Dot plot showing the expression level (colour intensity) and proportion of cells (dot size) expressing each HTO in each assigned sample. **C** Heatmap showing expression levels of each HTO tag in each assigned sample. **D** Violin plot showing the distribution of total RNA transcripts across sample assignments

In addition to standard scRNAseq data, scRNAbox can be used to analyze multiplexed samples, whereby each subject is tagged with a unique HTO, pooled, and then captured and sequenced together. Cell hashtagging can reduce the cost of scRNAseq by a factor of the number of samples multiplexed; however, additional steps are required to bioinformatically assign each cell back to its sample of origin. In Step 4, scRNAbox provides the option for users to demultiplex cells based on the expression of sample specific HTOs. To demonstrate, we analyzed a scRNAseq dataset of PBMCs from 8 subjects collected by Stoeckius et al. [18]. In Step 0, we selected to run the “HTO” analysis track and proceeded to run Steps 1–3 using the same analytical parameters that Stoeckius et al. used to process their data. At Step 4, instead of running doublet detection with DoubletFinder, scRNAbox uses the Seurat *MULTIseqDemux* function to assign cells back to their sample-of-origin based on HTO expression (19). ScRNAbox produces multiple figures to visualize the enrichment of HTOs across samples. Upon examining the expression levels of each HTO label across samples, we observed that cells with a distinct expression for a given HTO are assigned to the matching sample (Figs. 6A-C). Barcodes with multiple HTO labels are detected as doublets, as these likely represent two cells that were sequenced together. Negative cells have too low of a level of any HTO tag to be accurately assigned. We observed that the doublet group had about twice as many RNA transcripts per cell compared to the cells that were assigned to an individual sample, suggesting that the predicted doublets are likely true doublets (Fig. 6D**).** We conclude that scRNAbox pipeline can accurately demultiplex samples with HTO tags.

## Conclusions

Here, we introduce ScRNAbox, a comprehensive end-to-end pipeline designed to streamline the processing and analysis of single-cell transcriptomic data. ScRNAbox responds to the pressing demand for a user-friendly, HPC solution, bridging the gap between the growing computational demands of scRNAseq analysis and the coding expertise required to meet them. ScRNAbox empowers researchers, regardless of coding experience, to unlock the full potential of HPC clusters. By automating and optimizing the entire scRNAseq analysis workflow, it facilitates the processing of numerous samples while seamlessly scaling to meet user needs. The stepwise execution of ScRNAbox provides researchers with fine-grained control over parameters and manual cell annotations, ensuring reproducibility and customizability at every stage. The pipeline contains a rich array of functionalities, enabling cell type annotation, differential gene expression analysis, and efficient cell demultiplexing using Hashtag feature labels. In Table 3, we compare scRNAbox’s usability and capabilities to those of the most relevant scRNAseq analysis tools currently available.

**Table 3:** Comparison of scRNAbox’s usability and capabilities to the most relevant scRNAseq analysis tools currently available. Not available: the answer to this question remained uncertain after addressing the tool’s user manual and publication. Abbreviations: DGE, differential gene expression; HTO, oligonucleotide tagged Hashtag antibodies

	scRNAbox	CellRanger	Seurat (9)	Scanpy (10)	Asc-Seurat (2)	SC1 (3)	PIVOT (4)	ASAP (5)	NASQAR (6)	alona (7)	BingleSeq (8)
Usability											
Does it run on HPC automatically	Yes	No	No	No	No	No	No	No	No	No	No
Is it a web application	No	No	No	No	Yes	Yes	Yes	Yes	Yes	Yes	Yes
Easy to use by wet-lab biologists	Yes	Yes	No	No	Yes	Yes	Yes	Yes	Yes	Yes	Yes
Adaptable parameters	Yes	Yes	Yes	Yes	Yes	Yes	Yes	Yes	Yes	Yes	Yes
Outputs intermediate objects for bioinformaticians	Yes	Yes	Yes	Yes	No	Yes	No	No	Yes	No	Yes
Extensive detailed documentation	Yes	Yes	Yes	Yes	Yes	No	Yes	Yes	Yes	No	Yes
Generatates visualization and numeric outputs	Yes	Yes	Yes	Yes	Yes	Yes	Yes	Yes	Yes	Yes	Yes
Automatic pipeline to run all steps	Yes	Yes	No	No	No	No	No	No	No	Yes	No
Interactive pipeline to check each output	Yes	Yes	Yes	Yes	Yes	Yes	Yes	Yes	Yes	No	Yes
Data input											
Takes in FASTQ files	Yes	Yes	No	No	No	No	No	No	No	No	No
Processes multiple samples in parallel	Yes	Yes	No	No	No	No	No	No	No	No	No
Takes in count matrices (CellRanger outputs)	Yes	Yes	Yes	Yes	Yes	Yes	Yes	Yes	Yes	Yes	Yes
Takes in Seurat objects	Yes	No	Yes	No	No	No	No	No	No	No	No
Quality control and filtering											
Ambient RNA detection and adjustement	Yes	No	No	No	No	No	No	No	No	No	No
Doublet detection and filtering	Yes	No	Yes	Yes	No	No	No	No	No	Yes	No
Filtering by unique/total RNA	Yes	Yes	Yes	Yes	Yes	Yes	Yes	Yes	Yes	yes	Yes
Filtering by percent mitochondrial DNA	Yes	Yes	Yes	Yes	yes	Yes	No	Yes	Yes	No	No
Filtering by percent ribosomal DNA	Yes	Yes	Yes	Yes	No	Yes	No	Yes	Yes	No	No
Filter out gene lists	Yes	No	Yes	Yes	No	No	Yes	No	Yes	Yes	No
Filter out cell cycle genes	Yes	No	Yes	Yes	No	No	Yes	No	Yes	Yes	No
Regress out gene lists	Yes	No	Yes	Yes	No	No	No	No	Yes	No	No
Sample Integration											
Option to integrate samples	Yes	Yes	Yes	Yes	Yes	Yes	Yes	Not available	Yes	No	No
Option to integrate separatetely processed samples from independent experiements	Yes	Yes	Yes	Yes	No	No	No	No	No	No	No
Clustering											
Does clustering using Seurat	Yes	No	Yes	No	Yes	No	No	Yes	Yes	Yes	Yes
Allows tuning parameters for clustering and multple resolutions	Yes	Yes	Yes	Yes	Yes	Yes	Yes	Yes	Yes	Yes	Yes
Calculates adjusted rand index to select optimal clustering outputs	Yes	No	No	No	No	No	No	No	No	No	No
Cluster annotation											
Calculates intercluster DGE to find cluster markers	Yes	Yes	Yes	Yes	Yes	Yes	No	Yes	Yes	Yes	Yes
Gene enrichment analysis of cluster markers in cell type libraries	Yes	No	Yes	Yes	Yes	No	Yes	Yes	Yes	No	Yes
Calculates aggregated expression of user-defined gene sets	Yes	No	Yes	Yes	No	No	No	No	No	No	No
Predicts annotations with reference data	Yes	No	Yes	Yes	No	No	No	No	No	No	No
Allows visualization of custom marker sets	Yes	Yes	Yes	Yes	Yes	Yes	Yes	No	Yes	No	Yes
Differential gene expression											
Calculates DGE by user-defined clusters between user-defined variables	Yes	Yes	Yes	Yes	Yes	No	Yes	No	No	No	Yes
Provides pseudobulk DGE comparisons	Yes	No	Yes	Yes	No	No	No	No	No	No	No
HTO feature seq analysis											
Supports feature seq files	Yes	Yes	Yes	Yes	No	No	No	No	No	No	No
Provides HTO demultiplexing	Yes	Yes	Yes	Yes	No	No	No	No	No	No	No

*DGE*, Differential gene expression; *HTO*, oligonucleotide tagged Hashtag antibodies

While ScRNAbox offers an efficient solution for scRNAseq data analysis, it does come with certain limitations. Primarily tailored for sequencing alignment from 10X data and focused on differential gene expression analysis, ScRNAbox does not encompass trajectory analysis, cell-to-cell networks, or other downstream analytical methods. Nonetheless, it equips users with final and intermediate data objects that seamlessly integrate into external packages for advanced analyses.

Our open-source, modular code provides a versatile foundation for users to customize and expand. We encourage researchers to harness the flexibility of ScRNAbox, introducing alterations, additional options, or their preferred downstream analyses. With ScRNAbox, we aspire to simplify the intricacies of scRNAseq analysis, inviting an extended community of researchers to embark on novel and thoughtful explorations of single-cell transcriptomics.

## Supplementary Information

Supplementary Material 1

Supplementary Material 2

Supplementary Material 3

## Data Availability

Raw snRNAseq data of the post-mortem human midbrain prepared by Smajic et al. was obtained from the Gene Expression Omnibus (GEO) with accession number GSE157783. Raw scRNAseq data of human PBMCs prepared by Stoeckius et al. was obtained from the GEO with accession number GSE108313. Processed snRNAseq data of the post-mortem human midbrain prepared by Kamath et al. was obtained from the Single Cell Portal (https://singlecell.broadinstitute.org).

## Abbreviations

ARI: Adjusted rand index
DaN: Dopaminergic neurons
DEG: Differentially expressed genes
DGE: Differential gene expression
GO: Gene ontology
GSEA: Gene set enrichment analysis
GUI: Graphical user interface
HPC: High-performance computing
HTO: Oligonucleotide tagged Hashtag antibodies
L2FC: Log2 fold-change
OPC: Oligodendrocyte precursor cell
PBMC: Peripheral blood mononuclear cell
PC: Principal component
PCA: Principal component analysis
PD: Parkinson’s disease
scRNAseq: Single-cell RNA sequencing
snRNAseq: Single-nuclei RNA sequencing
UMAP: Uniform manifold approximation and projection
